# Case Report: Secondary syphilitic alopecia following non-penetrative sexual contact: a case of initial misdiagnosis

**DOI:** 10.3389/fmed.2026.1750303

**Published:** 2026-04-17

**Authors:** Yuyu Deng, Changxia Li, Lifang Cheng, Shaoheng Wang, Xiaohua Tao

**Affiliations:** 1 Dermatology Hospital of Jiangxi Province, Nanchang, China; 2 Jiangxi Provincial Clinical Research Center for Skin Diseases, Nanchang, China; 3 Candidate Branch of National Clinical Research Center for Skin Diseases, Nanchang, China; 4 Dermatology Institute of Jiangxi Province, Nanchang, China; 5 The Affiliated Dermatology Hospital of Nanchang University, Nanchang, China

**Keywords:** alopecia areata, misdiagnosis, non-penetrative sexual contact, secondary syphilis, syphilitic alopecia

## Abstract

We describe a case of secondary syphilitic alopecia following non-penetrative sexual contact that was initially misdiagnosed as alopecia areata. A 26-year-old unmarried male presented with moth-eaten patchy alopecia in the occipital region. Prior topical treatment for presumed alopecia areata at another facility was ineffective. The patient reported repeated episodes of mutual masturbation with a female massage worker two months earlier, and he noticed a small erythematous papule on the penile frenulum thereafter. Serological testing revealed a rapid plasma reagin (RPR) titer of 1:32 and a positive *Treponema pallidum* particle agglutination assay (TPPA), confirming syphilis. The final diagnoses were secondary syphilis and syphilitic alopecia. Treatment consisted of benzathine penicillin G, 2.4 million units intramuscularly weekly for 3 weeks. Within 3 months, complete hair regrowth was observed without scarring.

## Introduction

Syphilitic alopecia represents an uncommon manifestation of secondary syphilis, affecting approximately 3%−7% of patients (1). Typically appearing 3–6 months post-infection, it may present in moth-eaten, diffuse, or mixed patterns, often mimicking other alopecias such as alopecia areata. Although sexual transmission remains the primary route, transmission can occur via direct contact with infectious lesions during non-penetrative sexual activity or other intimate contact. We present a case of syphilitic alopecia initially misdiagnosed as alopecia areata, underscoring the importance of considering syphilis in patients with unexplained hair loss even when penetrative intercourse is denied.

## Case presentation

### History

A 26-year-old unmarried man presented with a 1-week history of patchy occipital alopecia, discovered incidentally during a haircut. He reported no pruritus, pain, or systemic symptoms. He had previously been diagnosed with alopecia areata at an outside institution and received topical therapy without improvement. The patient denied penetrative sexual intercourse but acknowledged multiple episodes of mutual masturbation with a massage worker approximately2 months prior. He also reported noticing a small red papule on the penile frenulum with mild erythema and slight swelling during that period. His past medical history was otherwise unremarkable.

### Physical examination

**Figure 1:**
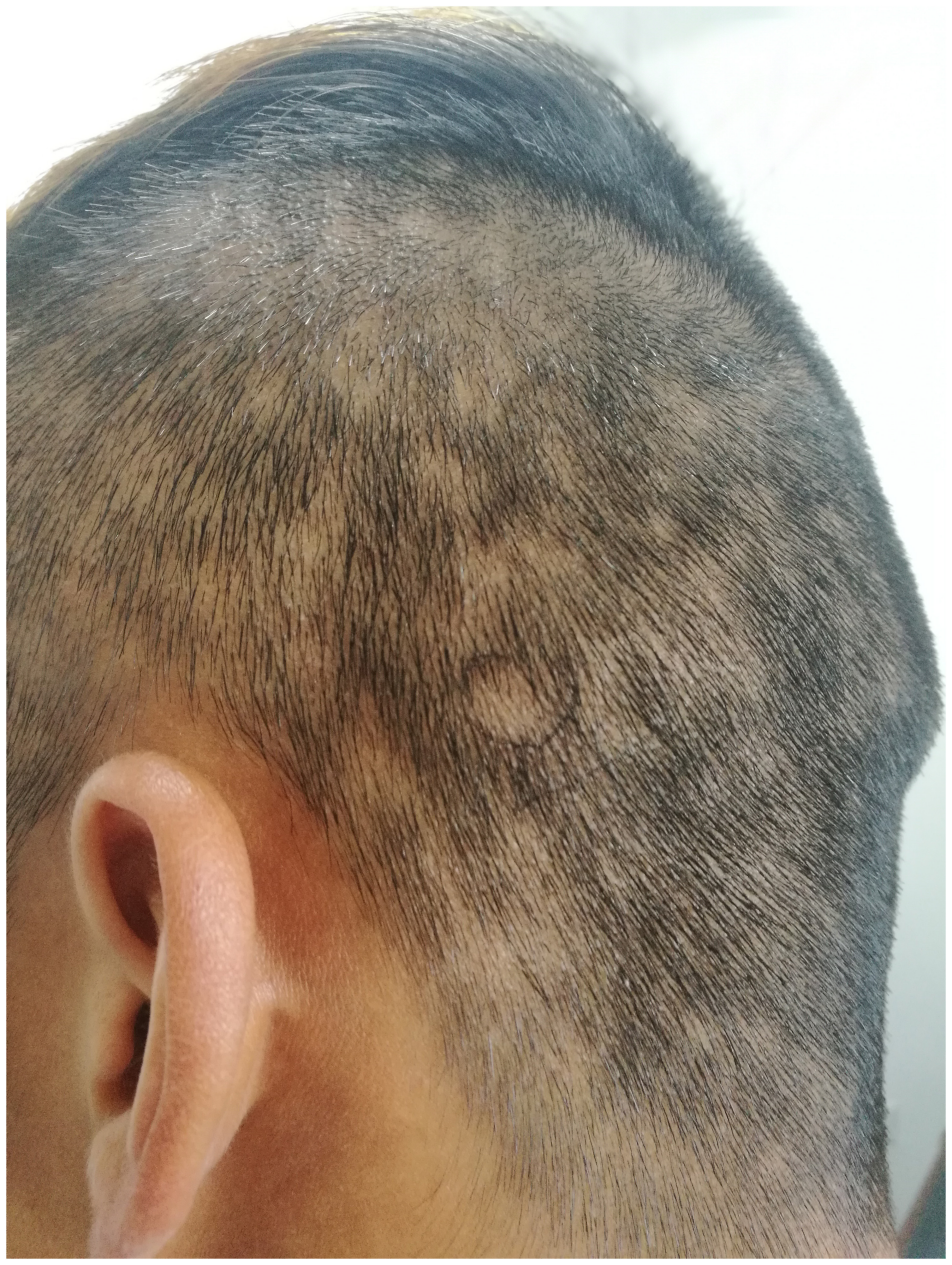
Worm-eaten type hair loss.

**Figure 2:**
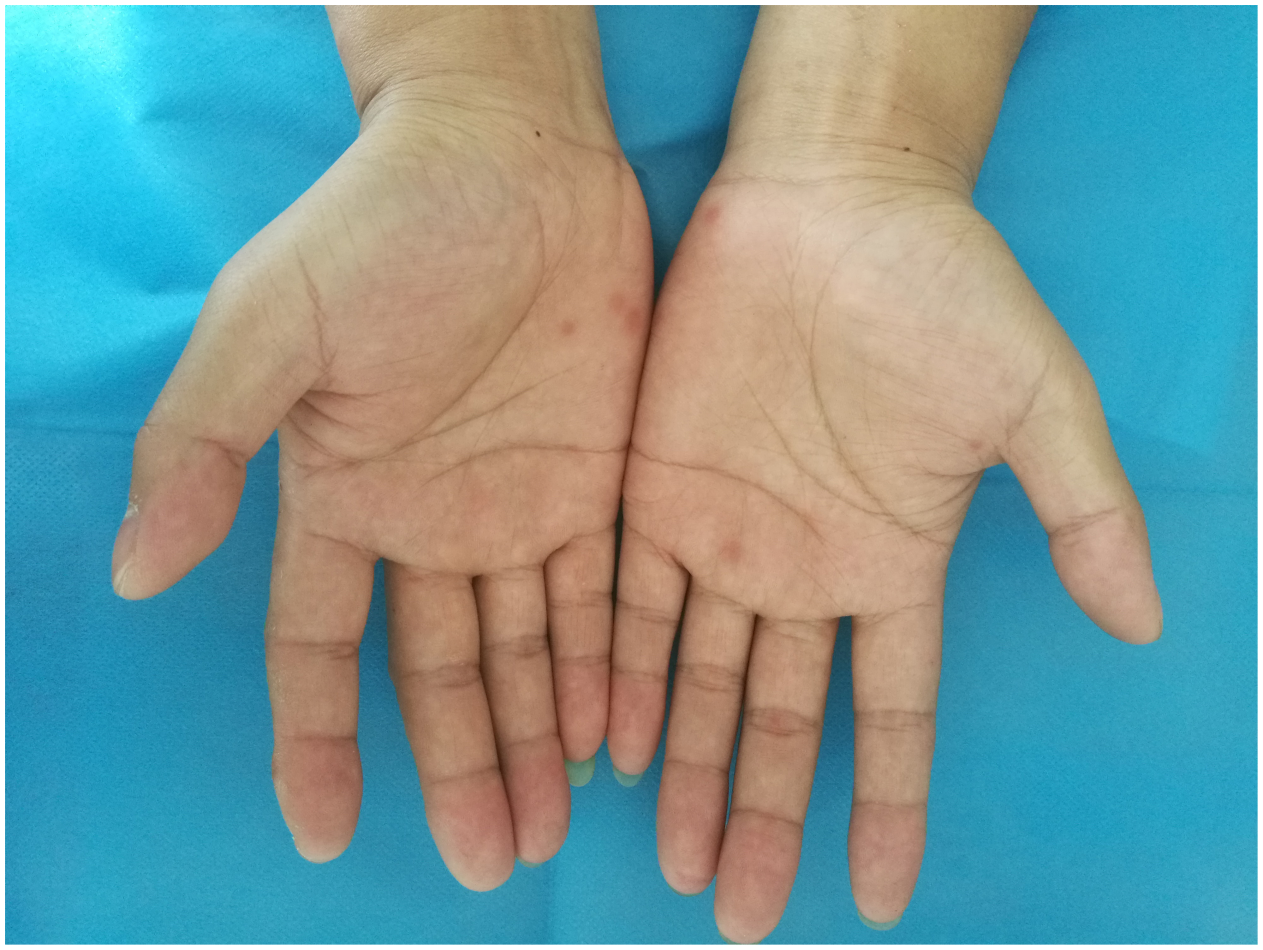
Copper-red maculopapular lesions were observed on both palms.

**Figure 3:**
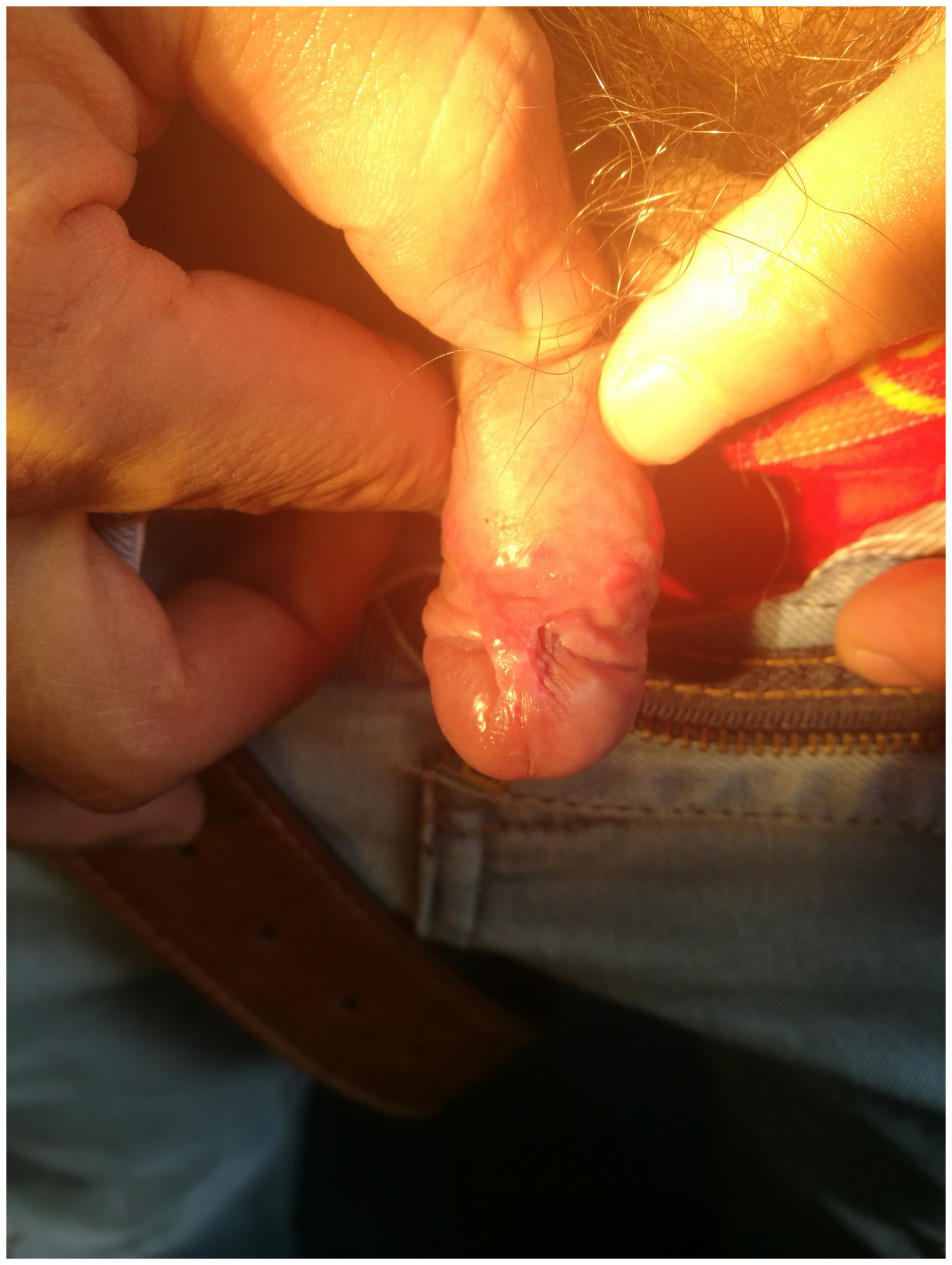
Genital examination identified a 0.5 cm dark-red maculopapule on the penile frenulum with superficial erosion, slight elevation, and minimal induration.

Palpable inguinal lymphadenopathy was present. Scalp examination revealed scattered punctate and patchy non-scarring alopecia with a characteristic moth-eaten pattern, without erythema, scaling, or scarring, and a negative hair pull test (Figure 1). Copper-red maculopapular lesions were observed on both palms (Figure 2), while the soles and trunk were unaffected. Genital examination identified a 0.5-cm dark-red maculopapule on the penile frenulum with superficial erosion, slight elevation, and minimal induration (Figure 3).

### Investigations

**Figure 4:**
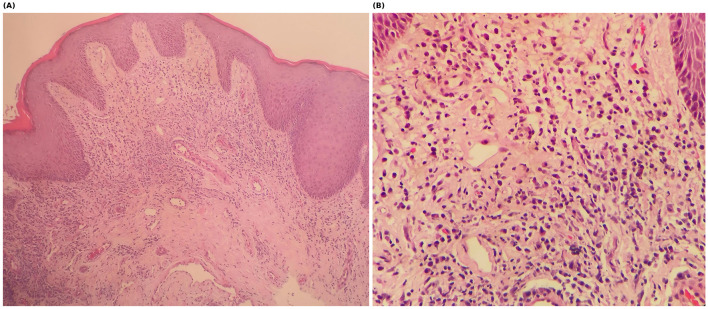
Histopathological findings of the penile lesion. **(A)** Low-power view (H&E, ×40) showing dense superficial-to-mid dermal perivascular inflammatory infiltrate. **(B)** Higher-power view (H&E, ×100) highlighting prominent plasma cells and vascular changes.

Serological testing confirmed syphilis infection with an RPR titer of 1:32 and positive TPPA. Screening tests for HIV, hepatitis B, and hepatitis C were negative. Histopathological examination of the scalp showed non-scarring alopecia without specific features of alopecia areata. No treponemal organisms were identified on routine sections [Option A: Treponemal immunohistochemistry/special stains (e.g., Warthin-Starry) were performed and were negative.] [Option B: Treponemal immunohistochemistry/special stains were not available/performed in our setting and therefore could not be evaluated.]. Biopsy of the penile lesion revealed mild epidermal hyperplasia with dense perivascular lymphoplasmacytic infiltration and numerous plasma cells, consistent with syphilitic infection (Figure 4). *T. pallidum* IHC was negative.

## Treatment and outcome

The patient received benzathine penicillin G, 2.4 million units intramuscularly weekly for 3 weeks. He experienced a transient Jarisch–Herxheimer reaction manifesting as low-grade fever and headache, which resolved spontaneously. The penile lesion healed within 2 weeks post-treatment. Hair regrowth commenced at 8 weeks, with complete restoration observed by 3 months. RPR titers declined to 1:8 at 3 months and 1:4 at 6 months, with TPPA remaining positive. At 1-year follow-up, the RPR was non-reactive and there was no evidence of recurrence. The massage worker was subsequently tested and found to have an RPR titer of 1:64 with positive TPPA.

## Discussion

This case highlights several important clinical considerations. First, it demonstrates that syphilis exposure can occur through non-penetrative sexual contact when infectious lesions are present. In this patient, the reported exposure consisted of mutual manual genital stimulation (hand-to-genital contact by both parties) without penetrative intercourse. The partner was subsequently found to have active syphilis serology. Although the partner's clinical stage at the time of exposure could not be confirmed due to the lack of a complete contemporaneous clinical evaluation, secondary syphilis is biologically plausible, as mucocutaneous lesions in secondary syphilis—particularly palmar eruptions—are highly infectious and may harbor abundant spirochetes. Therefore, repeated hand-to-genital contact could facilitate transmission via microabrasions or contact with infectious exudate. While the precise inoculation site cannot be proven, the temporal association, concurrent mucocutaneous findings, and concordant serology support the diagnosis of secondary syphilis with syphilitic alopecia in this case (2, 3).

The clinical presentation of syphilitic alopecia can be subtle. The moth-eaten pattern observed in our patient represents the most characteristic manifestation, though diffuse and mixed patterns also occur. Unlike alopecia areata, syphilitic alopecia typically lacks exclamation mark hairs and often shows predilection for the occipital region. The non-scarring nature and rapid response to appropriate therapy further support the diagnosis.

Histopathology may be variable. In our case, the scalp biopsy showed non-specific changes, whereas the penile lesion demonstrated the characteristic plasma cell-rich infiltrate supportive of syphilitic infection. This underscores the value of sampling an active mucocutaneous lesion and correlating pathology with serology. A limitation is that the partner's clinical findings at the time of exposure were not documented in detail; therefore, the exact inoculation source (hand vs. genital lesions) cannot be confirmed.

Alopecia is an uncommon manifestation of secondary syphilis, with a reported incidence of approximately 2.9%−11.2% in different series, and generally around 3%−7% (1, 4, 5). Syphilitic alopecia most often develops 3–6 months after infection, at which time the disease is typically in the secondary stage (6). Classically, syphilitic alopecia is divided into two types (7): (1) symptomatic alopecia, in which syphilitic cutaneous lesions (such as scaly papules) are present within the alopecic areas; this pattern is relatively rare; and (2) essential alopecia, in which there are no overt local skin lesions other than hair loss, and which represents the more common form. Essential syphilitic alopecia can be further subdivided into three clinical subtypes (8). The most typical is the moth-eaten pattern, characterized by multiple, small, irregularly marginated patches of alopecia scattered over the scalp, resembling areas “chewed out” by insects; hair is thinned but not completely absent, and the remaining hairs are of uneven length. Its hallmark features include non-scarring alopecia with no clinical signs of inflammation or significant scaling on the scalp, which constitute one of the key clues to secondary syphilis (9). The second subtype is diffuse alopecia (also referred to as “telogen effluvium–like” alopecia), presenting as generalized and even hair thinning that mimics telogen effluvium or diffuse alopecia and may be confused with androgenetic alopecia or true telogen effluvium (10). The third subtype is a mixed pattern, in which moth-eaten patches are superimposed on a background of diffuse hair thinning (11). Syphilitic alopecia has a predilection for the occipital and temporal regions, but may also involve the vertex as well as the eyebrows, eyelashes, and other body hair (8). In the present case, alopecia predominantly affected the occipital area and showed a classic moth-eaten pattern, which is consistent with previous reports. It should be emphasized that syphilitic alopecia does not destroy hair follicles, and the hair loss is reversible; with appropriate anti-syphilitic therapy, hair regrowth usually begins within approximately 8–12 weeks. This feature is helpful in distinguishing it from scarring alopecias, and also reminds clinicians to consider syphilis in cases of unexplained telogen effluvium.

## Conclusion

This case emphasizes that syphilis should remain in the differential diagnosis of unexplained alopecia, even when penetrative intercourse is denied. Comprehensive exposure history-taking, including non-penetrative sexual contact, coupled with appropriate serological testing, enables accurate diagnosis and prevents delays in treatment. Penicillin therapy remains highly effective, with an excellent prognosis for hair regrowth when instituted promptly. We also summarize key clinical features of syphilitic alopecia in Table 1.

**Table 1:** Characteristics of syphilis presenting predominantly with moth-eaten alopecia.

Item	Typical characteristics	Evidence
Sex	Predominantly affects males, though females can also be involved; the proportion of patients co-infected with HIV is not low.	Single-center data plus a systematic review show that most patients are male, with an HIV co-infection rate of approximately 56.5% in a 23-case cohort; female cases have also been reported (11).
Age	20–40 years old.	Retrospective studies report a mean age of 27.6 ± 8.8 years; a 5-case series reported ages from 31 to 46 years (11).
Disease course	Alopecia usually occurs within several weeks to months after disease onset; in most patients, significant hair regrowth is observed within 3 months after treatment.	One study proposed “alopecia occurring within 6 months of disease onset and marked regrowth within 6 months of treatment” as a diagnostic criterion; clinical follow-up frequently documents regrowth within about 3 months (11).
Pattern of alopecia	Producing a characteristic “moth-eaten” appearance; commonly involves the vertex and occipital regions; may affect eyebrows, beard, and body hair.	The “moth-eaten” pattern is the most common and suggestive type; the vertex–occipital area is most frequently affected.
Other clinical manifestations	Two patterns: (1) Symptomatic syphilitic alopecia (SA)—accompanied by secondary syphilitic lesions such as palmoplantar or truncal maculopapular eruptions, condyloma lata, and generalized superficial lymphadenopathy; (2) Essential SA—virtually no typical cutaneous lesions other than alopecia.	The distinction between these two patterns is consistent across multiple case reports; thinning of eyebrow, eyelash, axillary, and pubic hair has also been described.
Syphilis serology (RPR/TPPA)	Non-treponemal tests (e.g., RPR) are usually positive with medium-to-high titers; treponemal tests (TPPA/TPHA/FTA-ABS) are positive and used for confirmation and staging.	Reported RPR titers commonly fall within the 1:32–1:256 range; TPHA and FTA-ABS are typically positive.
Outcome and prognosis	Most patients achieve complete or near-complete hair regrowth following standard penicillin therapy.	Multiple case reports and case series have shown marked hair regrowth approximately 3 months after treatment (11).

## Data Availability

The original contributions presented in the study are included in the article/supplementary material, further inquiries can be directed to the corresponding author.
